# Assessment of Autistic Traits in Children Aged 2 to 4½ Years With the Preschool Version of the Social Responsiveness Scale (SRS‐P): Findings from Japan

**DOI:** 10.1002/aur.1742

**Published:** 2017-03-03

**Authors:** Andrew Stickley, Yoshiyuki Tachibana, Keiji Hashimoto, Hideyuki Haraguchi, Atsuko Miyake, Seiichi Morokuma, Hiroshi Nitta, Masako Oda, Yukihiro Ohya, Ayako Senju, Hidetoshi Takahashi, Takanori Yamagata, Yoko Kamio

**Affiliations:** ^1^ Department of Child and Adolescent Mental Health National Institute of Mental Health, National Center of Neurology and Psychiatry (NCNP) Kodaira Tokyo Japan; ^2^ Department of Human Ecology Graduate School of Medicine, University of Tokyo Bunkyo‐ku Tokyo Japan; ^3^ Stockholm Centre for Health and Social Change (SCOHOST) Södertörn University Huddinge Sweden; ^4^ Division of Infant and Toddler Mental Health Department of Psychosocial Medicine, National Medical Centre for Children and Mothers Setagaya‐ku Tokyo Japan; ^5^ Division of Rehabilitation Medicine and Developmental Evaluation Centre National Centre for Child Health and Development (NCCHD) Setagaya‐ku Tokyo Japan; ^6^ Research Center for Environment and Developmental Medical Sciences, Kyushu University Fukuoka Japan; ^7^ National Centre for the Japan Environment and Children's Study National Institute for Environmental Studies Tsukuba Ibaraki Japan; ^8^ Faculty of Life Science Kumamoto University Kumamoto Japan; ^9^ Division of Allergy Department of Medical Sciences, National Center for Child Health and Development (NCCHD) Setagaya‐ku Tokyo Japan; ^10^ Japan Environment and Children's Study UOEH Subunit Center, University of Occupational and Environmental Health Fukuoka Japan; ^11^ Department of Pediatrics Jichi Medical University Tochigi Japan

**Keywords:** ASD, autistic traits, quantitative measure, reliability, validity, preschool children, questionnaire

## Abstract

The recent development and use of autism measures for the general population has led to a growing body of evidence which suggests that autistic traits are distributed along a continuum. However, as most existing autism measures were designed for use in children older than age 4, to date, little is known about the autistic continuum in children younger than age 4. As autistic symptoms are evident in the first few years, to address this research gap, the current study tested the preschool version of the Social Responsiveness Scale (SRS‐P) in children aged 2 to 4½ years in clinical (*N* = 74, average age 40 months, 26–51 months) and community settings (*N* = 357, average age 39 months, 25–50 months) in Japan. Using information obtained from different raters (mothers, other caregivers, and teachers) it was found that the scale demonstrated a good degree of internal consistency, inter‐rater reliability and test‐retest reliability, and a satisfactory degree of convergent validity for the clinical sample when compared with scores from diagnostic “gold standard” autism measures. Receiver operating characteristic analyses and the group comparisons also showed that the SRS‐P total score discriminated well between children with autism spectrum disorder (ASD) and those without ASD. Importantly, this scale could identify autistic symptoms or traits distributed continually across the child population at this age irrespective of the presence of an ASD diagnosis. These findings suggest that the SRS‐P might be a sensitive instrument for case identification including subthreshold ASD, as well as a potentially useful research tool for exploring ASD endophenotypes. ***Autism Res** 2017, 10: 852–865*. © 2017 International Society for Autism Research, Wiley Periodicals, Inc.

## Introduction

Autism spectrum disorder (ASD) comprises a group of neurodevelopmental conditions characterized by the presence of stereotypical, restricted behaviors, and impaired communication and social interaction skills [American Psychiatric Association, 2000, 2013]. Research has revealed that this condition is common around the world, with a recent epidemiologically rigorous study from South Korea finding a prevalence of 2.6% [Kim et al., 2011], and that rather than simply being either present or absent, ASD is a quantitative phenomenon that falls along one end of a continuum of impairment, distributed across the general population [Constantino, 2011; Curran et al., 2011; Hoekstra, Bartels, Verweij, & Boomsma, 2007; Posserud, Lundervold, & Gillberg, 2006]. In particular, evidence suggests that a broader autism phenotype is observed in the family members of individuals diagnosed with autism [Bailey, Palferman, Heavey, & Le Couteur, 1998; Constantino, Zhang, Frazier, Abbacchi, & Law, 2010; Lyall et al., 2014; Piven, Palmer, Jacobi, Childress, & Arndt, 1997] and that ASD symptoms aggregate at a sub‐clinical level that stretches beyond pervasive developmental disorder not‐otherwise specified (PDD‐NOS) [Kamio, Inada, et al., 2013]. These research findings converge in a direction which suggests that gaining an understanding of individual differences in autistic traits in non‐clinical populations, as well as among individuals with ASD, will be key to determining the etiology of autism [Lundström et al., 2012; Robinson et al., 2011].

This evolving understanding of ASD has been facilitated by assessment tools such as the Autism‐Spectrum Quotient—Children's Version [AQ‐Child; Auyeung, Baron‐Cohen, Wheelwright, & Allison, 2008], Autism Spectrum Screening Questionnaire [ASSQ; Posserud et al., 2006], Childhood Autism Spectrum Test [CAST; Williams et al., 2008], and Social Responsiveness Scale [SRS; Constantino & Gruber, 2005] that have been developed and used in the general population for research purposes. The use of these quantitative measures, all of which target autistic symptoms/traits in children aged 4 years and above, has also enhanced the quick identification of children with ASD.

As yet, however, very little is known about the autistic continuum in children under 4 years old. This is not only because there are very few parent questionnaires that rate a range of ASD symptoms that can be widely distributed and easily completed to determine social impairment in this age range, but also, because there has been a paucity of research on the prevalence of ASD in preschool children [Nygren et al., 2012]. This may be due to the fact that children are more likely to be diagnosed with ASD after age 4, even though a diagnosis of ASD can be made as early as age 2 and remains stable during toddlerhood [Chawarska, Klin, Paul, Macari, & Volkmar, 2009]. The Centers for Disease Control and Prevention [2014] recently reported that the median age of the first ASD diagnosis remains above 4, while research from elsewhere has shown that two‐thirds of ASD cases identified in the mainstream school population are still undiagnosed and untreated [Kim et al., 2011].

The absence of information on ASD symptoms in the general population under age 4 is an important research gap, with potentially large societal costs, especially as the severity of social impairment when as young as age 3 might be a predictor of long‐term outcomes in adulthood among individuals with autism [Howlin, Moss, Savage, & Rutter, 2013]. The notion that autistic impairment is a quantitative trait [Constantino, 2011], and the recognition of the public health importance of decreasing the age when children are first diagnosed with ASD, and enrolled in community‐based support systems [Centers for Disease Control and Prevention, 2014], has highlighted the need for assessment tools that can accurately quantify autistic symptoms/traits of individuals at this young age.

Despite this, most early ASD screeners such as the Checklist for Autism in Toddlers [Baron‐Cohen, Allen, & Gillberg, 1992], the Modified Checklist for Autism in Toddlers [Robins, Fein, Barton, & Green, 2001], or the Early Screening of Autistic Traits Questionnaire [Dietz, Swinkels, van Daalen, van Engeland, & Buitelaar, 2006] focus on toddlers at 18–24 months of age, and ASD‐specific information for children aged 3 years in the community remains sparse. However, one measure that has been developed in recent years which is relevant in this context is the preschool version of the SRS (SRS‐P) for 3‐year‐old children [Pine, Luby, Abbacchi, & Constantino, 2006]. This is a modified form of the standard SRS for children aged 4–18 years [Constantino & Gruber, 2005], and is almost identical to the SRS‐2 Preschool Form, its current version [Constantino & Gruber, 2012]. The standard SRS is sensitive to autistic symptoms or traits in children, even in subthreshold ASD conditions [Kamio, Inada, et al., 2013 ], while the inter‐individual variation in the degree of autistic traits assessed by it seems highly preserved over time [Constantino et al., 2009]. For these reasons, it has been widely used for research purposes, such as in genetic epidemiological research [Constantino, Hudziak, & Todd, 2003; Reiersen, Constantino, Volk, & Todd, 2007], research assessing brain‐behavior relationships [Noriuchi et al., 2010], and for detecting autism‐related genetic loci [Duvall et al., 2007].

Besides its quickness and ease of use, previous research has shown that the SRS is a valid quantitative measure of autistic traits when compared to “gold standard” autistic assessment measures such as the Autism Diagnostic Interview Revised (ADI‐R) [Constantino, Davis, et al., 2003]. Moreover, its utility as a screener has been reported for both general and clinical populations, although the ROC results seem sample‐dependent. The original SRS manual recommends the use of a cut‐off score of 70 for boys (65 for girls) with a sensitivity of 0.77 and a specificity of 0.75 for the purpose of primary screening in the low‐risk general population. When a cut‐off of 75 was used among typically developing children in Germany, the sensitivity was 0.80 and specificity was 1.0 [Bölte, Westerwald, Holtmann, Freitag, & Poustka, 2011]. Similarly, the SRS was found to have excellent discriminant validity for Mexican schoolchildren with a score of 60 for the parent rating and 59 for the teacher rating providing an optimal trade‐off between sensitivity and specificity [Fombonne, Marcin, Bruno, Tinoco, & Marquez, 2012]. In a U.S. study parent and teacher rated cut‐off scores of 60 and 54 were respectively chosen to maximize sensitivity and specificity in discriminating ASD children from non‐affected siblings [Schanding, Nowell, & Goin‐Kochel, 2012]. In studies in clinical children a parent‐rated score of 75 identified ASD with a sensitivity of 0.80–0.85 and a specificity of 0.69–0.75 against non‐ASD clinical cases (attention‐deficit/hyperactivity disorder (ADHD), unspecific developmental disorder, other child psychiatric diagnoses) [Bölte et al., 2011; Constantino & Gruber, 2005]. For Taiwanese preschool cases, a mixed raters' score of 65 that resulted in a sensitivity of 0.94 and a specificity of 0.70 was suggested as an optimal cut‐off for screening, while a score of 87 which had a sensitivity of 0.66 and a specificity of 0.90 was judged as being optimal for clinical classification [Wang, Lee, Chen, & Hsu, 2012]. As for U.K. children with special educational needs, a parent‐rated score of 75 identified ASD with a sensitivity of 0.78 and a specificity of 0.67, although low IQ and behavior problems reduced specificity [Charman et al., 2007]. When used in a tertiary level clinic in Australia, a parent‐rated T‐score of 60 identified ASD with a sensitivity of 0.91 and a specificity of 0.08, while a teacher‐rated T‐score of 60 had a sensitivity of 0.84 and a specificity of 0.42 [Aldridge, Gibbs, Schmidhofer, & Williams, 2012]. By increasing the cut‐off scores to 89.5 for the parent form, and to 70.0 for the teacher form, the specificity increased to 0.92 for parent scores, and 0.83 for teacher scores. Among a mixed Japanese sample that included ASD, non‐ASD clinical and typical developing children, a parent‐rated score of 53.5 for boys and 52.5 for girls was suggested as being optimal for the purpose of primary screening for case referral and 109.5 for boys and 102.5 for girls for the purpose of secondary screening for diagnostic classification [Kamio, Inada, et al., 2013], while teacher‐rated scores of 58.0 for boys (sensitivity 0.725, specificity 0.667) and 43.0 for girls (sensitivity 0.789, specificity 0.667) were judged as being optimal screening cut‐off scores [Kamio, Moriwaki, & Inada, 2013]. These previous studies have shown that the SRS can be regarded as a valid screening tool across different cultural settings, although purpose‐specific cut‐off scores need to be chosen.

In contrast, to the best of our knowledge, there has been only one standardization study of the preschool version on 247 U.S. children aged 2^1^/_2_ to 4^1^/_2_ [Constantino & Gruber, 2012], where Asian children were underrepresented. In addition, psychometric and validation evidence for children at this age is less intensive. Given the comparative lack of autistic symptoms/traits assessment tools that can be quickly completed for very young children, the aim of the current study was to provide psychometric and validation evidence for the SRS‐P in Asian children, which has never before been reported. This will build on and extend earlier research conducted in the United States which used the SRS‐P [Pine et al., 2006; Turner‐Brown, Baranek, Reznick, Watson, & Crais, 2013].

This study reports the reliability and validity of the SRS‐P to quantitatively assess the degree of autistic symptoms or traits in Japanese children aged 2 to 4^1^/_2_ years old drawn from a clinical sample with and without ASD, as well as a non‐clinical community sample.

## Methods

### Study Participants

Two groups of children took part in the current study. The first “community” group (*N* = 357, [200 males, data were missing on sex for one child]) who were recruited from geographically different communities in Japan in 2008 and 2013. The majority of children (*N* = 279 [78.2%]) came from a pilot study [Kawamoto et al., 2012] of a large‐scale on‐going longitudinal birth cohort study (the Japan Environment and Children's Study: JECS) [Kawamoto et al., 2014] which has been designed to examine the effects of environmental exposures on health from birth to 13 years of age, while the rest (*N* = 78) were recruited via community kindergartens or nurseries. The characteristics of the JECS participants (singleton births, gestational age at birth, sex, and birth weight) and maternal age were similar to those found in national survey data on the Japanese general population [Michikawa et al., 2015]. Families' household income [Michikawa et al., 2015] was also similar to the national average reported by the Japanese Ministry of Health, Labour and Welfare (http://www.mhlw.go.jp/english/database/db-hss/dl/report_gaikyo_2011.pdf). In terms of the current study all of these children were assumed to be representative of the general child population.

The second “clinical” group (*N* = 74) consisted of children recruited from affiliated research centers with specialized clinics for developmental disorders (the National Center of Neurology and Psychiatry [NCNP] and the National Center for Child Health and Development [NCCHD]) in Tokyo. This group consisted of 40 children diagnosed with ASD (the ASD group, 23 males) and 34 children with non‐ASD neuropsychiatric diagnoses (the non‐ASD group, 21 males). The clinical diagnoses of ASD and its subcategories were confirmed by a research team that included experienced child psychiatrists and licensed clinical psychologists according to DSM‐IV‐TR criteria based on all available clinical information. To corroborate each diagnosis, we evaluated the severity of autistic symptoms using the Japanese versions of the ADI‐R [Tsuchiya et al., 2013] and the Autism Diagnostic Observation Schedule (ADOS) [Kuroda et al., 2013] administered by research‐reliable interviewers. The ASD group consisted of 26 children with Autistic Disorder (AD), 10 children with PDD‐NOS, one child with Asperger's Disorder, and 3 children unspecified. These three children (all females, normal range IQs) could have been allocated to either the PDD‐NOS or Asperger's Disorder category but agreement could not be reached about the specific subcategory. The non‐ASD group contained 22 outpatients of a pediatric rehabilitation clinic who had documented diagnoses such as Down syndrome, lysosomal storage disease, spina bifida, head trauma, and sequelae of acute encephalitis, that were made by a clinical team including pediatric neurologists, speech therapists, occupational therapists, and clinical psychologists. In addition, 12 children who were initially suspected of having ASD at health check‐ups, who were referred to the NCNP, but were found not to have the condition after a full evaluation, were included in the non‐ASD group (developmental delay, suspected ADHD, oppositional defiant disorder, specific phobia, obsessive‐compulsive disorder, enuresis, tic disorder, reactive attachment disorder). The sex ratio did not significantly differ between the groups (*χ* ^2^ = 0.40, *P* = 0.81). Regarding age, there was no significant group difference between boys (*U* = 3239.00, *P* = 0.09), although girls in the clinical group were significantly older than girls in the community group (40.93 months vs. 38.41, *U* = 1583.00, *P* = 0.01). There was no significant age difference between girls in the ASD and non‐ASD groups (*t* (28) = 0.18, *P* = 0.86). Details of the study participants are presented in Table 1.

**Table 1:** Characteristics of the Study Participants

	Clinical group (*N* = 74)						Community group (*N* = 357)		
Characteristic	ASD			Non‐ASD					
	Male	Female	Total	Male	Female	Total	Male	Female	Total
Sex (*N*)^a^	23	17	40	21	13	34	200	156	357
Average age (months) (SD)	40.44 (6.14)	41.11 (6.85)	40.73 (6.38)	40.14 (4.81)	40.69 (5.53)	40.35 (5.02)	38.63 (3.35)^b^	38.41 (3.57)^c^	38.52 (3.45)^d^
[Range]	[33–51]	[26–49]	[26–51]	[34–50]	[35–50]	[34–50]	[35–47]	[25–50]	[25–50]
Developmental level (N [%])									
Average range	13 [56.5%]	7 [41.2%]	20 [50.0%]	7 [33.3%]	5 [38.5%]	12 [35.3%]			
Borderline	1 [4.3%]	1 [5.9%]	2 [5.0%]	5 [23.8%]	1 [7.7%]	6 [17.6%]			
Mild developmental delay	6 [26.1%]	7 [41.2%]	13 [32.5%]	5 [23.8%]	4 [30.8%]	9 [26.5%]			
Moderate developmental delay	2 [8.7%]	2 [11.8%]	4 [10.0%]	3 [14.3%]	2 [15.4%]	5 [14.7%]			
Severe developmental delay	1 [4.3%]	0 [0.0%]	1 [2.5%]	1 [4.8%]	1 [7.7%]	2 [5.9%]			
DQ/IQ (SD)	82.58 (17.41)^e^	72.76 (19.68)^f^	78.93 (18.63)^g^	79.25 (20.70)^h^	83.63 (22.40)^i^	80.71 (20.89)^j^			
[Range]	[47–103]	[49–117]	[47–117]	[30–115]	[38–110]	[30–115]			
ADI‐R total (Mean [SD])	26.12 (6.50)^k^	25.53 (10.86)^l^	25.87 (8.50)^m^	9.00 (8.89)^n^	7.50 (0.71)^o^	8.40 (6.35)^p^			
[Range]	[15–36]	[10–48]	[10–48]	[2–19]	[7–8]	[2–19]			
ADOS total (Mean [SD])	12.59 (4.40)^k^	13.35 (5.23)^k^	12.97 (4.78)^q^	3.33 (1.53)^n^	3.00 (4.24)^o^	3.20 (2.39)^p^			
[Range]	[5–21]	[2–21]	[2–21]	[2–5]	[0–6]	[0–6]			
ADOS CSS (Mean [SD])	6.38 (1.78)^r^	6.23 (3.06)^s^	6.31 (2.39)^t^						
[Range]	[2–9]	[1–10]	[1–10]						

a Information on sex was missing for one child in the community sample. ^b^Calculated for 175 participants. ^c^Calculated for 152 participants. ^d^Calculated for 328 participants. ^e^Calculated for 22 participants. ^f^Calculated for 13 participants. ^g^Calculated for 35 participants. ^h^Calculated for 16 participants. ^i^Calculated for 8 participants. ^j^Calculated for 24 participants. ^k^Calculated for 17 participants. ^l^Calculated for 13 participants. ^m^Calculated for 30 participants. ^n^Calculated for 3 participants. ^o^Calculated for 2 participants. ^p^Calculated for 5 participants. ^q^Calculated for 34 participants. ^r^Calculated for 16 participants. ^s^Calculated for 13 participants. ^t^Calculated for 29 participants.

The developmental levels of participants in the clinical group, that were clinically judged based on standardized assessment and/or administrative records, ranged widely from average to severe developmental delay, although normal range including borderline level was most common in both the ASD and non‐ASD groups. Regarding the distribution across developmental levels, there was no statistical difference between boys and girls in either the ASD (*P* = 0.79 [Fisher's Exact Test]) or the non‐ASD group (*P* = 0.84). When boys and girls were combined, there was no significant difference between the ASD and non‐ASD group (*P* = 0.33). Standardized developmental assessment information (DQ or IQ) was available for 59 children (35 ASD children and 24 non‐ASD children). The most common measure used was the Kyoto Scale of Psychological Development (KSPD) [Society for the Kyoto Scale of Psychological Development Test, 2008], which is comparable to the Bayley Scales of Infant Development second edition [Bayley, 1993]. For the rest, IQs assessed using the Tanaka‐Binet Intelligence Scale V for children or the Wechsler Preschool and Primary Scale of Intelligence (WPPSI) were available. Since the KSPD DQ in children with ASD is comparable to an IQ [Koyama, Osada, Tsujii, & Kurita, 2009], both DQs and IQs were combined for the analyses in this study. There were no significant sex differences in IQ/DQ scores in either the ASD (*t* [33] = 1.53, *P* = 0.13) or the non‐ASD group (*t* [22] = −.48, *P* = 0.64). When boys and girls were combined, there was also no significant difference between the two clinical groups (*t* [57] = −0.34, *P* =0.73).

### SRS‐P Assessment Procedure

For the community group, SRS‐P ratings were provided by mothers (*N* = 328) and/or teachers (*N* = 73), with 44 of these children being rated by both mothers and teachers. For the clinical group, ratings were obtained from mothers (*N* = 72) and/or fathers (*N* = 32)/grandmothers (*N* = 3) (hereafter termed “other caregivers”) as well as teachers/childcare workers (*N* = 33) (hereafter termed “teachers”). Among them, 33 children were rated by both mothers and other caregivers, 31 children were rated by both mothers and teachers, and 33 children were rated by both other caregivers and teachers. In addition, for 32 children in the clinical group, across‐time SRS‐P ratings were obtained from mothers at two separate time points in 2012–2013. The average time interval between the two tests was 15.2 days (SD 8.3, range 3–51 days).

### Measures

#### Autistic symptoms/traits

The current study makes use of an initial preschool version of the SRS designed for use in 3‐year olds (SRS‐P). The preschool and standard versions of the test are almost identical in terms of content except for the use of different wordings to describe behavior (14 items) that is appropriate in groups with different ages. When items of the SRS‐P are compared to those in the current SRS‐2 Preschool Form, they are almost identical except for tiny changes in 24 items (“his/her” in the SRS‐P to “his or her” in the SRS‐2 Preschool, “toys” in the SRS‐P to “a toy” in the SRS‐2 Preschool, “emotion” in the SRS‐P to “feeling” in the SRS‐2 Preschool). Like the original SRS and the SRS‐2 Preschool Form, the SRS‐P consists of 65 items with answers ranging from “not true” (scored 0) to “almost always true” (scored 3) to give a total score ranging from 0 to 195, with higher scores indicating a greater degree of impairment, taking parents or teachers between 15 and 20 minutes to complete.

The SRS‐P asks about behavior in the previous 3 months, although the original SRS and the SRS‐2 Preschool Form ask about the last 6 months [Constantino & Gruber, 2012]. The Japanese language adaptation was conducted by members of our research team with permission from Western Psychological Services (WPS). The Japanese translation was created to ensure consistency with the standard version, and was back‐translated into English by independent translators, and then scrutinized for content equivalence by the scale developer (J.C.). The developers and WPS then approved the final Japanese version, which we used in this study. As a result, the Japanese version of the SRS‐P differed from that of the current SRS‐2 Preschool Form only in the expression of item 33 which was simpler in the SRS‐P and by targeting behavior in the previous 3 months instead of 6 months. Regarding the gold standard ASD measures based on professionals' observation, we used total scores of three domains of the Japanese version of the ADI‐R [Tsuchiya et al., 2013] and total scores of the Social and Communication domains of the Japanese version of ADOS [Kuroda et al., 2013] for the analysis in this study. Since the use of Calibrated Severity Scores (CSS) has been shown to be more valid as an indicator of autism severity than the ADOS raw total score [Gotham, Pickles, & Lord, 2009; Schumwzy et al., 2012], CSS scores calculated from raw ADOS scores [Gotham et al., 2009, 2008] were used when examining validity in the subsequent analyses.

### Statistical Analyses

SRS‐P raw scores were used in all analyses. Correlation analyses (Pearson's *r*) were used to examine the relation between SRS‐P scores and age and IQ/DQ. In addition, to better understand the effects of age, we also examined if there were differences in scores between children aged ≥ 36 months and those aged < 36 months using *t*‐tests, a Mann–Whitney *U*‐test, and linear regression analysis. SRS‐P scores from different raters were compared with the use of the Mann Whitney *U*‐test for the community sample, while a one‐way Analysis of Variance (ANOVA) or Kruskal–Wallis test was used to compare mean scores for the clinical sample depending on the normality of the distribution of the scores.

The reliability of the SRS‐P scores was examined in three ways. First, the *internal consistency* of the 65 items for the community and clinical samples was tested by computing the Cronbach alpha (*α*) statistic. *Inter‐rater reliability* was examined by using the intraclass correlation coefficient (ICC) for the total score. The same tests were used to examine the *test‐retest reliability* of mother ratings across time in the clinical group for the SRS‐P total score.

Validity was also examined in three ways. The *convergent validity* was assessed by correlating the total SRS‐P scores with the ADI‐R score using both the “ever” and “current” score, and the ADOS CSS score for the ASD group. Further, the *discriminant validity* of the SRS‐P total scores was examined with *t*‐tests for independent samples or the Mann–Whitney *U*‐test depending on the normality of the scores distribution. Finally, a receiver operating characteristic (ROC) analysis was also performed on the entire sample to determine how well the mother and teacher‐rated SRS‐P scores distinguished children with ASD in terms of establishing an appropriate cut‐off score.

## Results

### SRS‐P Scores

The mean total SRS‐P scores, their standard deviations (SD) and the range of the scores are presented in Table 2. Using the mother's ratings, there was no association between the total SRS‐P score and age (months) in either the community (*r* = −0.026) or the clinical group (*r* = 0.023). Although the majority of children were over 36 months old, stratifying the mothers' ratings by age band (≥ 36 months vs. < 36 months) showed that the score of children aged < 36 months did not differ significantly from those aged 36 months or above for the whole clinical group (*t*[70] = 0.447, *P* = 0.657), either ASD children (*t*[38] = 0.898, *P* = 0.375), or non‐ASD children (*t*[30] = −0.708, *P* = 0. 484), and for children in the community sample (*U* = 0.5356.50, *Z* = −0.928, *P* = 0.353) (see Supporting information, Table S1). Linear regression analyses to further examine the association between age and mothers' SRS‐P ratings using age as a continuous variable revealed that there was no association for all children in the clinical sample (Beta [*B*] = 0.023, *P* = 0.850), for ASD children (*B* = −0.009, *P* = 0.957), or non‐ASD children (*B* = 0.042, *P* = 0.818), separately. Although there was a negative correlation between the total SRS‐P score and IQ/DQ in both the ASD (*r* = −0.502) and the non‐ASD (*r* = −0.475) groups (mother's ratings) as well as for the whole clinical group (*r* = −0.476 [*N* = 58]), when restricted to children with normal IQ/DQ, the correlations were non‐significant in both the ASD (*r* = −0.125) and the non‐ASD (*r* = −0.323) groups (mother's ratings, data not shown in the table) as well as for the whole clinical group (*r* = −0.196 [*N* = 38]).

**Table 2:** Scores on the SRS‐P for Children in the Clinical and Community Group

	Clinical group (*N* = 74)			Community group (*N* = 357)	
	All children	ASD	Non‐ASD	
*Rater*	Mean (SD)	Mean (SD)	Mean (SD)	Mean (SD)
	[Range: Min–Max]	[Range: Min–Max]	[Range: Min–Max]	[Range: Min–Max]
*Mother*	62.63 (27.08)^a^	70.45 (25.83)^b^	52.84 (25.74)^c^	35.93 (15.74)^d^
	[11–139]	[19–139]	[11–96]	[9–106]
*Other caregiver*	58.60 (29.72)^e^	72.00 (32.07)^f^	50.68 (25.80)^g^	
	[7–139]	[23–139]	[7–105]	
*Teacher*	52.36 (24.03)^h^	71.64 (26.06)^i^	42.73 (16.28)^j^	36.99 (19.38)^k^
	[15–128]	[36–128]	[15–73]	[7–93]

a Calculated for 72 participants. ^b^Calculated for 40 participants. ^c^Calculated for 32 participants. ^d^Calculated for 328 participants. ^e^Calculated for 35 participants. ^f^Calculated for 13 participants. ^g^Calculated for 22 participants. ^h^Calculated for 33 participants. ^i^Calculated for 11 participants. ^j^Calculated for 22 participants. ^k^Calculated for 73 participants.

In the community group the mean SRS‐P total score rated by mothers (35.9) and by teachers (37.0) was similar with no significant difference (*U* = 11842.50, *Z* = −0.145 *P* = 0.885). In the clinical group, a one‐way ANOVA test showed that there was no difference between the different raters' total scores: *F*(2, 137) = 1.634, *P* = 0.199, with a Scheffé post‐hoc test revealing no difference between mother and other caregiver scores (*P* = 0.772), mother and teacher scores (*P* = 0.201) or between other caregiver and teacher scores (*P* = 0.639). There was also no significant difference in the mean scores by rater in the ASD group (*F*[2, 61] = 0.020, *P* = 0.980), while the same result was obtained for the scores in the non‐ASD group (*P* = 0.381 [Kruskal–Wallis test]).

Thus, the mean SRS‐P scores rated by mothers were independent of age (months) in this age range, independent of DQ/IQ within the normal range but inversely related to DQ/IQ for the entire range of DQ/IQ scores, and there were no significant differences in the mean SRS‐P scores among different raters in either the community or the clinical group.

### Internal Consistency

There was a high level of internal consistency for the scores obtained from the 65 individual SRS‐P items in both the community and the clinical group with Cronbach's alpha falling below 0.9 in only one instance—for mother ratings in the community group (0.891, [*N* = 328]) (Table 3).

**Table 3:** Internal Consistency of Raters' Scores on the SRS‐P for Children in the Clinical and Community Group

	Clinical group (N = 74)			Community group (N = 357)	
*Rater*	All children	ASD	Non‐ASD		
Mother	.942^a^	.934^b^	.941^c^	.891^d^
Other caregiver	.954^e^	.960^f^	.941^g^	
Teacher	.941^h^	.930^i^	.915^j^	.922^k^

a Calculated for 72 participants. ^b^Calculated for 40 participants. ^c^Calculated for 32 participants. ^d^Calculated for 328 participants. ^e^Calculated for 35 participants. ^f^Calculated for 13 participants. ^g^Calculated for 22 participants. ^h^Calculated for 33 participants. ^i^Calculated for 11 participants. ^j^Calculated for 22 participants. ^k^Calculated for 73 participants.

### Inter‐Rater Reliability

For the community group, only the mother's and the teacher's scores could be compared. The ICC for this was modest and of borderline significance (ICC = 0.234, *P* = 0.064 [*N* = 44]). All ICCs for the clinical group were statistically significant at the *P* < 0.05 level (Table 4). This indicates that there was a good level of accordance between different raters' scores in the clinical group with better agreement for ASD children than non‐ASD children, although confidence intervals were fairly wide for some of the point estimates.

**Table 4:** Inter‐rater Reliability for Scores on the Preschool Social Responsiveness Scale (SRS‐P) for Children in the Clinical Group (*N* = 74) Assessed with Intraclass Correlation Coefficients

Raters association	All Children	ASD	Non‐ASD
Mother–other caregiver	0.740 (0.534–0.863)^a^	0.895 (0.696–0.967)^b^	0.561 (0.160–0.801)^c^
Mother–teacher	0.544 (0.243–0.750)^d^	0.504 (−0.076–0.835)^e^	0.480 (0.085–0.751)^f^
Other caregiver–Teacher	0.616 (0.351–0.789)^g^	0.706 (0.242–0.910)^h^	0.448 (0.067–0.722)^i^

*Note*. Figures in parentheses 95% confidence intervals.

a Calculated for 33 participants, *P* < 0.001. ^b^Calculated for 13 participants, *P* < 0.001. ^c^Calculated for 20 participants, *P* = 0.005. ^d^Calculated for 31 participants, *P* = 0.001. ^e^Calculated for 11 participants, *P* = 0.047. ^f^Calculated for 20 participants, *P* = 0.008. ^g^Calculated for 33 participants, *P* < 0.001. ^h^Calculated for 11 participants, *P* = 0.005. ^i^Calculated for 22 participants, *P* = 0.012.

### Test‐Retest Reliability

It was also possible to examine the test‐retest reliability of the SRS‐P using data from the mothers of 32 children in the clinical group (ASD 13, non‐ASD 19). This showed that there was a very high level of concordance for the overall SRS‐P score across time for the whole sample and for children with and without ASD. Specifically, the ICC score for all children in the clinical group was 0.915 (95% CI: 0.832–0.957), for ASD children it was 0.920 (95% CI: 0.766–0.975) and for non‐ASD children it was 0.904 (95% CI: 0.771–0.962).

### Convergent Validity

To determine how the SRS‐P scores converge on the “gold standard” autism measures such as the ADI‐R or ADOS, the correlations were examined for 30 children diagnosed with ASD who were fully assessed using these measures. Mother‐rated SRS‐P total scores were correlated with total ADI‐R scores to a strong degree (*N* = 30, *r* = 0.741, *P* < 0.01) and the relation was almost unchanged when the scores were replaced by the current ones (*N* = 30, *r* = 0.729, *P* < 0.01), while they were correlated with the total ADOS CSS scores to a moderate degree (*N* = 29, *r* = 0.430, *P* = 0.020).

### Discriminant Validity

The SRS‐P ratings from mothers were compared between groups using Mann–Whitney *U*‐tests. This showed that there were highly significant differences between the community and the ASD group (*U* = 1650.0, *Z* = −7.73, *P* < 0.001) as well as between the community and the clinical groups (*U* = 4905.0, *Z* = −7.77, *P* < 0.001). A comparison of mothers' ratings between the ASD and non‐ASD groups showed that the scores of ASD children were significantly higher than those of non‐ASD children (70.45 > 52.84; *t* [70] = 2.87, *P* = 0.005). Large and significant differences were also obtained when comparing teacher ratings between the community and the ASD group (*U* = 2807.5, *Z* = −3.91, *P* < 0.001), between the community and the clinical groups (*U* = 732.0, *Z* = −.3.23, *P* = 0.001), and between the ASD and the non‐ASD groups (*t* [31] = 3.92, *P* < 0.001).

**Figure 1:**
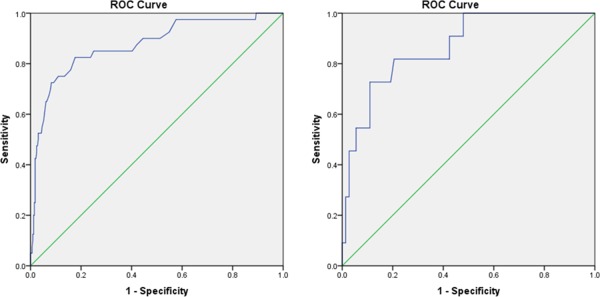
(**A**) ROC curve for the mother‐rated SRS‐P scores for ASD children in the clinical group and those for community children. (**B**) ROC curve for the teacher‐rated SRS‐P scores for ASD children in the clinical group and community children.

**Figure 2:**
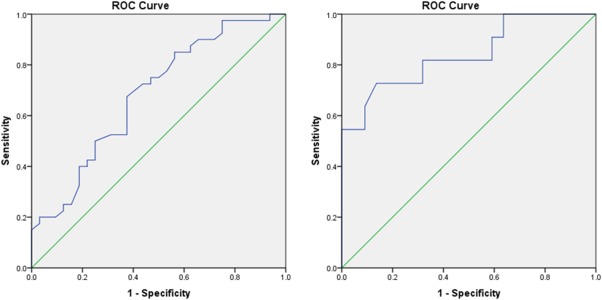
(**A**) ROC curve for the mother‐rated SRS‐P scores for ASD and non‐ASD children in the clinical group. (**B**). ROC curve for the teacher‐rated SRS‐P scores for ASD and non‐ASD children in the clinical group.

ROC analyses of the mother ratings revealed the full extent of the scale's ability to distinguish children with ASD against community children with an area of 0.874 under the curve (AUC) (95% CI: 0.810–0.939) (see Fig. 1A). A score of 48.5 on the SRS‐P had a sensitivity of 0.825 and a specificity of 0.823 when it is used as a primary screening tool. Teacher ratings also distinguished well between ASD children and community children (AUC = 0.867, 95% CI: 0.762–0.973) with a cut‐off score of 51.5 having a sensitivity of 0.818 and specificity of 0.795 (Fig. 1B). More variability was observed, however, in the scale's performance when used to compare ASD and non‐ASD children within the clinical group (Fig. 2A,B). For teachers' scores (*N* = 33) the scale distinguished well between these children (AUC = 0.841, 95% CI: 0.687–0.995), with a score of 51.5 having a sensitivity of 0.818 and a specificity of 0.682. However, the AUC for mothers' ratings (*N* = 72) was lower at 0.681 (95% CI: 0.557–0.806). A score of 46.5 had a sensitivity of 0.825 and a specificity of 0.438, while a score of 70.5 resulted in sensitivity reducing to 0.500 but specificity increasing to 0.750. When these cut‐off points were used to classify children against a gold standard measure—ADOS (total communication and social interaction scores) in clinical children who had both SRS and ADOS ratings, the percentage of children who exceeded the cut‐offs of both measures was 69.2% (27/39), and 38.5% (15/39) for mother's ratings of 46.5 and 70.5, respectively, and 70.0% (7/10) for the teacher's rating when a score of 51.5 was used. Mothers' ratings distinguished better between children who were classified as having ASD according to ADOS and those who were not when compared to a clinical ASD diagnosis based on DSM‐IV‐TR (AUC = 0.817, 95% CI: 0.643–0.991, sensitivity 0.871, specificity 0.750 for 46.5 as a cut‐off score; sensitivity 0.484, specificity 0.875 for 70.5 as a cut‐off score). The discriminative ability of teachers' ratings was however, poor in relation to the ADOS classification (AUC = 0.313, 95% CI: 0.000–0.686, sensitivity 0.875, specificity 0.000), possibly due to the small sample size (*N* = 10).

**Figure 3:**
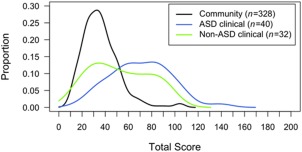
Distribution of the preschool version of the Social Responsiveness Scale (SRS‐P) raw scores in the community and clinical (ASD and non‐ASD) groups.

The mother‐rated SRS‐P scores for children in the community and clinical (ASD and non‐ASD) groups are plotted graphically in Fig. 3. While covering almost the entire spectrum of scores, the community, ASD and non‐ASD clinical children had distinct distributions, with the peak of the distribution much higher up the scale for the ASD children compared to both the community and non‐ASD children—although the overlap among the three groups reinforces the notion that autistic symptoms or traits assessed with this scale are distributed continually across non‐clinical and clinical populations irrespective of the presence of an ASD diagnosis, as emphasized by the fact that within the clinical group only 2/32 mother‐rated scores in the non‐ASD group fell outside the range of scores for the ASD group.

## Discussion

This study examined the psychometric properties of the SRS‐P for Japanese children aged 25–51 months recruited from community and clinical settings. The results of this study indicated that the SRS‐P measures autistic symptoms or traits in young children in this age range in both community and clinical samples with or without ASD in a reliable manner and has a clinical utility, a finding which has also been observed for the standard version of the scale [Aldridge et al., 2012; Bölte, Poustka, & Constantino, 2008; Constantino, Przybeck, Friesen, & Todd, 2000; Constantino, Davis, et al., 2003; Constantino, Hudziak, et al., 2003; Constantino et al., 2007; Fombonne et al., 2012; Kamio, Inada, et al., 2013; Kamio, Moriwaki, et al., 2013; Kanne, Abbacchi, & Constantino, 2009; Schanding et al., 2012; Wang et al., 2012]. Japanese community children (mother ratings 35.9, teacher ratings 37.0) scored lower than U.S. children (parents ratings 40.7, teacher ratings 42.8) across this age range [Constantino & Gruber, 2012], a result which replicates an earlier finding observed among schoolchildren [Kamio, Inada, et al., 2013]. The distribution of SRS‐P scores for children in this age range with and without ASD overlapped in a similar manner to the way it did in a previous study on schoolchildren [Kamio, Inada, et al., 2013], and thus provides further evidence for the continuous nature of autistic symptoms or traits in the above and below diagnostic threshold child population.

The internal consistency, inter‐rater and test‐retest reliabilities in the community and clinical samples were, in general, very good, although the size of the confidence intervals for some scores indicated variability in the precision of some estimates. It was noticeable, however, that the degree of correlation between mother and teacher ratings for the community sample was somewhat less than satisfactory, which is consistent with the universal pattern seen across these kind of clinical measures including all SRS‐2 forms, of higher correlational scores in clinical than in normative samples [Constantino & Gruber, 2012]. It is uncertain what underpins this mother‐teacher discordance given the close correspondence between these raters' mean scores, although it is possible that mothers and teachers of non‐clinical children were highlighting different aspects of social behavior as being problematic, whereas mothers and teachers of children with any neuropsychiatric problems at this age may identify social problems in a similar way. This suggests that as with the standard SRS, parent reports and teacher reports should be used separately and gathered together until future research clarifies this issue, which seems consistent with findings from the U.S. [Schanding et al., 2012] and the Netherlands [Duvekot, van der Ende, Verhulst, & Greaves‐Lord, 2015]. The high degree of correlation in raters' scores across time was in keeping with the finding from an earlier study for preschool children with an average age of 49 months across a longer interval [Pine et al., 2006].

There were moderate to large correlations between the SRS‐P scores and the scores obtained from “gold standard” autism measures, i.e., the ADI‐R and ADOS CSS. The magnitude of the correlation with the ADI‐R was in accord with the result from Pine et al.'s (2006) study that used the SRS‐P, and findings from studies using the standard SRS for older children [Charman et al., 2007; Constantino, Davis, et al., 2003; Kamio, Inada, et al., 2013] but higher than that observed in the Bölte et al. (2008) study. The magnitude of the correlation with the ADOS was also in keeping with the finding from Pine et al.'s (2006) SRS‐P study, and with those seen in studies utilizing the standard SRS [Bölte et al., 2008; Charman et al., 2007; Constantino et al., 2007; Kamio, Inada, et al., 2013]. Since the ADI‐R and ADOS were designed for categorical classification rather than dimensional assessment, such a close association between autistic symptoms assessed by these instruments with different methodologies also provides support for the validity of the SRS‐P [Constantino & Gruber, 2012]. In our sample, both mother and teacher SRS‐P ratings could discriminate between ASD children and those with other neuropsychiatric disorders, as well as non‐clinical children with a moderate degree of accuracy.

Since the initial development of the standard SRS [Constantino & Gruber, 2005], a growing body of research evidence from around the world has highlighted its cross‐cultural validity as an instrument to quantitatively assess autistic symptoms or traits in schoolchildren [Aldridge et al., 2012; Bölte et al., 2008; Fombonne et al., 2012; Jussila et al., 2015; Kanne et al., 2009; Schanding et al., 2012; Wang et al., 2012; Wigham, McConachie, Tandos, Le Couteur; Gateshead Millennium Study core team, 2012]. Although a preschool version (the SRS‐P) was subsequently designed, it was not widely disseminated. It was only very recently that a (slightly modified) version of it has become publicly available for use in children aged 2½ to 4½ [Constantino & Gruber, 2012].

The psychometric properties found for the SRS‐P from the present study, mentioned above, concur with those from earlier studies that have used the standard SRS with schoolchildren in different country settings. Specifically, the capacity that the SRS‐P displayed in this study as an instrument to assess autistic symptoms or traits mirrors that seen in an earlier Japanese study of children aged 6–15 years old [Kamio, Inada, et al., 2013]. Moreover, the differing distribution of the scores between community, ASD and non‐ASD clinical children that fell along a continuum but with a different center of gravity also mirrors the results seen earlier for Japanese schoolchildren [Kamio, Inada, et al., 2013]. This suggests that even at age 2 to 4½, autistic symptoms or traits are a clearly discernible phenomenon that is continuous and can be reliably and validly quantified, although the scale's diagnostic validity remains to be examined.

It should be noted that many children in our non‐ASD clinical group had relatively high scores overlapping with the score range of the ASD group. In part this might be explained by the fact that some of the children who were eventually included in the non‐ASD group were initially suspected of having ASD but later found not to meet the criteria. Nevertheless, this finding of a behavioral overlap between cases that are classified as distinct entities when using traditional diagnostic boundaries may provide further evidence for the common co‐occurrence of autistic symptoms in young neuropediatric patients. Indeed, the aggregation of autistic traits measured by quantitative ASD scales such as the SRS, has also been reported for youth and adults with neuropsychiatric disorders [Kamio, Inada, et al., 2013; Matsuo et al., 2015; Pine, Guyer, Goldwin, Towbin, & Leibenluft, 2008; Towbin, Pradella, Gorrindo, Pine, & Leibenluft, 2005]. Such a behavioral overlap seems to run parallel with recent evidence implying a possible etiological and pathophysiological overlap between ASD and non‐ASD disorders. A population‐based study of 9‐ and 12‐year‐old twin pairs revealed that ASD and non‐ASD neurodevelopmental disorders such as ADHD, tic disorders, and developmental coordination disorders seem to have a common genetic etiology [Lichtenstein, Carlström, Råstam, Gillberg, & Anckarsäter, 2010]. Further, a recent genome‐wide analysis demonstrated that specific single nucleotide polymorphisms are associated with a range of childhood‐onset and adult‐onset psychiatric disorders such as ASD, ADHD, bipolar disorder, major depressive disorder, and schizophrenia, suggesting the pleiotropic effects of variation in calcium‐channel activity genes on cross‐disorder psychopathology [Cross‐Disorder Group of the Psychiatric Genomics Consortium, 2013].

The behavioral overlap observed in the present study between ASD and non‐ASD cases might also however, be exaggerated since several studies have pointed out that non‐ASD factors such as general behavioral problems, or cognitive ability may exert an influence on the SRS scores [Charman et al., 2007; Frazier et al., 2014; Hus, Bishop, Gotham, Huerta, & Lord, 2013]. For clarification of the wider distribution of autistic symptoms/traits across traditionally different clinical entities, further clinically valid evidence is necessary, which might be beneficial also for understanding the fundamental mechanisms contributing to a broad vulnerability to developmental psychopathology.

Given the growing body of evidence on the benefits of early interventions for children with ASD [Camarata, 2014; Warren et al., 2011] and evidence (albeit, somewhat limited, and still too sparse) that these interventions may have a long‐term effect [Matson & Konst, 2013], this research has a clear clinical implication. As many cases of ASD continue to go unrecognized and untreated, there is an obvious need for better detection of ASD during the preschool period at the population level. In particular, as this scale is able to detect subthreshold autistic symptoms it might be especially important. This feature of the SRS‐P might also facilitate its use in autism research for identifying early biomarkers.

One of the major limitations of this study is that having a small sample size may have resulted in less precise estimates. In particular, the low number of children in the clinical group might help explain why there were fairly large confidence intervals observed for some of the point estimates. In addition, due to the small size of the clinical sample, data from boys and girls were not examined separately, although there were no significant differences in the sex ratio of any of the groups. Future research will thus need to clarify sex differences in the distribution of scores, reliability and validity of this scale for this age range. Second, the non‐ASD clinical group was comprised of children with neurological disorders with known genetic causes and children with idiopathic behavioral/developmental disorders. Further evaluation of the clinical validity of the SRS‐P in different clinical settings would be helpful for clinicians to choose the optimal instrument and the cut‐off scores. Third, this study did not collect sociodemographic information except gender and age, and information on general behavioral problems, parental stress, and IQ/DQ scores was not available for many children with severe or profound intellectual disabilities. In general, non‐ASD clinical factors such as general behavioral problems need to be considered in the interpretation of the SRS data, although previous research has indicated that ADHD and anxiety diagnoses seem less likely to substantially contaminate autism symptom measurement in non‐ASD siblings older than 4 years old [Frazier et al., 2014]. Fourth, since this study did not collect any autism‐related information (measurements) other than the SRS‐P scores for the community group, it is not known whether outside the clinical setting low scoring children actually had fewer autistic traits than high scoring children. Lastly, all the ASD cases had been diagnosed and families were aware of the child's condition when they completed the scale. Whether families who were not aware of their child's condition would rate it in a similar or different way should be evaluated in future research to determine the value of this scale as an ASD primary screening tool.

## Supporting information


**Table S1**. Scores on the Preschool Social Responsiveness Scale (SRS‐P) for children in the clinical and community sample by age group
