# Supplementary material for: Assessment of Autistic Traits in Children Aged 2 to 4½ Years With the Preschool Version of the Social Responsiveness Scale (SRS‐P): Findings from Japan
Source: Autism Res. 2017 Mar 3;10(5):852–65. doi: 10.1002/aur.1742 (PMC6586029; doi:10.1002/aur.1742)
Supplement: Supplementary file 1 — Table S1. Scores on the Preschool Social Responsiveness Scale (SRS‐P) for children in the clinical and community sample by age group [file AUR-10-852-s001.docx]

**Table 1A** Scores on the Preschool Social Responsiveness Scale (SRS-P) for children in the clinical and

community sample by age group

|  |  | Clinical Group (N = 74) Community Group  (N = 357) | | | | |
| --- | --- | --- | --- | --- | --- | --- |
|  | ***All Children*** | ***ASD*** |  | ***Non-ASD*** |  |  |
|  |  |  |  |  |  |  |
|  | Mean (S.D.) | Mean (S.D.) |  | Mean (S.D.) |  | Mean (S.D.) |
|  | [Range: Min-Max] | [Range: Min-Max] |  | [Range: Min-Max] |  | [Range: Min-Max] |
|  |  |  |  |  |  |  |
| ***Mother*** $\boldsymbol{\geq}$36 months | 62.01 (26.75)^a^ | 68.75 (25.95)^b^ |  | 54.07 (25.90)^c^ |  | 35.61 (15.57)^d^ |
|  | [11-139] | [19-139] |  | [11-96] |  | [9-106] |
| < 36 months | 66.00 (29.96)^e^ | 78.42 (25.58)^f^ |  | 44.25 (26.32)^g^ |  | 38.17 (16.89)^h^ |
|  | [21-104] | [33-104] |  | [21-82] |  | [12-101] |

Calculated for: ^a^ 61 participants; ^b^ 33 participants; ^c^ 28 participants; ^d^ 287 participants; ^e^ 11 participants; ^f^ 7 participants;

^g^ 4 participants; ^h^ 41 participants.
